# Emulating complex simulations by machine learning methods

**DOI:** 10.1186/s12859-021-04354-7

**Published:** 2021-11-12

**Authors:** Paola Stolfi, Filippo Castiglione

**Affiliations:** Institute for Applied Computing, National Research Council of Italy, Rome, Italy

**Keywords:** Type-2 diabetes, Emulation, Computational modelling, Risk prediction, Self-assessment

## Abstract

**Background:**

The aim of the present paper is to construct an emulator of a complex biological system simulator using a machine learning approach. More specifically, the simulator is a patient-specific model that integrates metabolic, nutritional, and lifestyle data to predict the metabolic and inflammatory processes underlying the development of type-2 diabetes in absence of familiarity. Given the very high incidence of type-2 diabetes, the implementation of this predictive model on mobile devices could provide a useful instrument to assess the risk of the disease for aware individuals. The high computational cost of the developed model, being a mixture of agent-based and ordinary differential equations and providing a dynamic multivariate output, makes the simulator executable only on powerful workstations but not on mobile devices. Hence the need to implement an emulator with a reduced computational cost that can be executed on mobile devices to provide real-time self-monitoring.

**Results:**

Similarly to our previous work, we propose an emulator based on a machine learning algorithm but here we consider a different approach which turn out to have better performances, indeed in terms of root mean square error we have an improvement of two order magnitude. We tested the proposed emulator on samples containing different number of simulated trajectories, and it turned out that the fitted trajectories are able to predict with high accuracy the entire dynamics of the simulator output variables. We apply the emulator to control the level of inflammation while leveraging on the nutritional input.

**Conclusion:**

The proposed emulator can be implemented and executed on mobile health devices to perform quick-and-easy self-monitoring assessments.

## Background

Mathematical modelling has allowed the development of highly detailed models describing phenomena in virtually all scientific fields. Such fidelity has been reached through the development of complex models such as fully non-linear ordinary or partial differential equations, multivariate latent factor models, agent-based models, often combined. These models are used to numerically simulate phenomena that can not be easily observed through real-world experiments due to economical, practical, or even ethical reasons. It comes alone that such models need to be an accurate representation of the reality to derive meaningful conclusions. Such precision, however, is paid in terms of computational cost. With the purpose to overcome this limitation, emulators are statistical models whose aim is to reproduce the main aspects of the dynamics produced by a simulation tool. The literature related to emulators provides solutions mainly related to (i) extrapolation of model outputs with respect to generic inputs (e.g. [1, 2]), (ii) the optimisation of objective functions depending on the output (e.g. [3]), and (iii) the tuning of parameters to fit real data (e.g. [4]). In particular, Bayesian methods have shown success in predicting deterministic functions realising the input/output map of specified computer simulations. In this framework, it is assumed that the output of the computer algorithm *y* is the mean of a random process *Y*, hence the random process represents a kind of knowledge regarding *y*. Such knowledge is specified through priors on the parameters of *Y*, although, most of the time, one has zero knowledge and therefore such priors have to be chosen by means of a cross-validation procedure. One of the most used techniques in this regard is the so-called *Gaussian process regression*, also known as Kriging in the field of geostatistics [5]. The strength of the Bayesian approach is that posterior distributions of the parameters provide a measure of the prediction uncertainty, that is, given a vector of input values it is possible to predict the output variable and the corresponding confidence interval at a given confidence level; the size of the confidence interval is a measure of the prediction uncertainty that can help to reshap the experimental design of critical input values. This approach has been investigated in [6] where it has been proposed a posterior entropy criterion for finding optimal designs on multidimensional grids. Indeed, this criterion is based on the fact that the smaller the posterior entropy the better the prediction. The main issues with the Bayesian approach in the field of emulation are that its computational cost depends on the dimension of the priors’ parameters, which in turn is related to the dimension of the input of the simulation model. Moreover, the estimation of the posterior distribution requires the inversion of a generally-large covariance matrix whose dimension is proportional to the dimension of the sample. An up to date review in this field is provided by [7], where a round up of the principal techniques is presented, namely, linear regression, support vector regression, Gaussian processes regression and mixtures of them. That article also provides an overview of surrogate models performances, (i) when used for global and local optimisation, (ii) when they need to satisfy some constraints, (iii) when used for sampling strategies focusing on stationary and adaptive ones. Recently, other techniques from the field of machine learning have been considered for the emulation purpose. In [8] an artificial neural network has been used as an emulator, with two sampling methods, the first based on a pure adaptive strategy aiming at reducing the predicted variance, the second based on a mix of space-filling and adaptive strategies. Since the artificial neural network does not provide an estimated prediction variance, as Gaussian processes do, it has been computed using *jackknife resampling* technique, which consists in training repeatedly the model by resampling one observation each time. In [9] a random forest has been used to investigate the behaviour of agent-based models and an adaptive sampling technique has been proposed to improve the prediction ability of the algorithm. It has been pointed out that the main advantage of using a random forest as an emulator is the high interpretability of its output, something that is instead lost when using, for instance, support vector regression. The proposed adaptive sampling is based on the idea of iteratively selecting data to add to a starting training set having more prediction uncertainty. In [10] we used a random forest to predict and analyse the impact of the input variables on the dynamics of a complex multi-scale simulation model, being a mixture of ordinary differential equations and agent-based modelling, able to predict the risk of type-2 diabetes (T2D). The mentioned computational model (herein referred to as M-T2D) has been implemented to take into account a set of user input data and to subsequently provide an estimation of the risk to develop a T2D clinical picture. In particular, given anthropometric parameters such as age, sex, body weight, height, and providing nutritional habits, fitness status and physical activity patterns by the user, the M-T2D calculates the risk of progressing toward a T2D-related state in a predefined time horizon.

That study, [10], was restricted to predic the final value of the dynamics in a long simulation and to identify the most relevant features. In the present work, we aim at extending the prediction by “emulating” the whole dynamics. This work is worthwhile mainly for two reasons. While the first goal is related to the possibility of “enacting” a cheaper computation on mobile devices for real-time assessment of the risk of T2D, the second is to challenge a machine learning methodology by using it as an emulator of a highly complex multi-output computational model. In this regard, the present study can provide an example of emulating a complex biological model so to make feasible its implementation on mobile health devices. The value of having a pre-trained emulator which is executable on mobile devices is to be found in the arising interest toward precision medicine, indeed it could facilitate the development of personalised treatment of diabetes risk of each patient on an individual basis [11]. This work is thus better valued looking at the increasing development of self-monitoring systems nowadays embedded in portable communication devices which opens up to the application of predictive tools in health care [11]. The work described in [12] is a first attempt of this kind which uses the *one step ahead* approach for emulating a complex model. Here we consider another approach which shows better predicting capabilities. To summarise, together with [12], the present study provides an example of use of machine learning techniques to emulate complex simulations.

## The simulation model

In this section, we briefly discuss the simulation tool (herein referred to as M-T2D) that we aim to emulate. We describe the output generated and underline the complexity of the simulation to reveal how challenging is attempting to emulate its dynamics.

M-T2D consists of a multi-level patient-specific model able to integrate metabolic, nutritional, and lifestyle data for the prediction of the metabolic and inflammatory processes underlying the development of T2D in the absence of familiarity [13]. It can be shortly described as a whole-body model for fuel homeostasis including metabolic, hormonal and inflammatory changes due to physical exercise and food ingestion [14, 15] and consisting of a combination of ordinary differential equation and agent-based modelling. M-T2D includes several model compartments: (i) a model of energy balance and weight gain/loss [16], based on the equations provided by [17] and [18]; (ii) the emergence of the inflammation is described as the result of adipose mass increase which, in turn, is a direct consequence of a prolonged excess of high-calorie intake [19]; (iii) a model for the anti-inflammatory mechanisms promoted during exercise by the skeletal muscles [20]; (iv) a model describing the inflammatory status of the subject by means of a general-purpose simulator of the immune system [21] (a modelling framework used to study different human pathologies [22, 23], and also aspects of non-human immunity [24]).

**Table 1:** The different virtual subjects have been generated by varying the parameters in this table and corresponding to 46170 different initial conditions

Anthropometric measures	
$\bullet$ Sex $S\in \{female, male\}$	
$\bullet$ Age $A\in \{28, 38, 48, 58, 68\}$	
$\bullet$ Weight $W\in \{ underweight, normal, overweight \}$	
$\bullet$ Height $H\in \{ short, average, tall \}$	
Physical activity	
$\bullet$ Number of sessions per week $N_{\mathrm{PA}} \in \{ 0, 1, 2, 3\}$	
$\bullet$ Duration (mins) $D_{\mathrm{PA}} \in \{ low=30, medium=60, high=90 \}$	
$\bullet$ Intensity (% of VO_2max_) $I_{\mathrm{PA}} \in \{ low = 40, high = 60 \}$	
Food intake (3 meals per day, breakfast, lunch, dinner)	
$\bullet$ Carbohydrates (grams) $C_{\mathrm{ME}} \in \{ low, med, high \}$	
$\bullet$ Proteins (grams) $P_{\mathrm{ME}} \in \{ low, med, high \}$	
$$\bullet$$ Fats (grams) $$F_{\mathrm{ME}} \in \{ low, med, high \}$$

**Table 2:** Coverage probability of $95\%$ bootstrap confidence bands

	Sample 1	Sample 2	Sample 3	Sample 4
$\beta$	0.70	0.78	0.80	0.81
$\gamma$	0.89	0.89	0.90	0.90
$\tau$	0.64	0.66	0.67	0.68

### The generation of synthetic data

Simulated trajectories of the dynamical model M-T2D starting from different initial conditions (i.e., consisting of anthropometric features, physical activity patterns and dietary habits) corresponding to different virtual subjects have been generated by varying the parameters reported in Table 1. There are $K=46170{}$ possible combinations of the input variables defining the characteristic of the virtual individual that are shown in Table 1.

The complete patient specification of the initial condition of the simulation is given as a string vector. For instance, the initial condition specified by the string female 28 obese tall 2 60/40 low/high/low corresponds to a 28 years old female subject, tall and obese, which exercises twice a week (sixty minutes each time with an intensity of 40%VO_2max_) and which follows a diet consisting in a low amount of carbohydrates and fats but rich in proteins. We indicate the vector corresponding to the initial condition as follows:

1 $$\begin{aligned} {\varvec{x}}=\big [ S,A,W,H,(N_{PA},D_{\mathrm{PA}},I_{\mathrm{PA}}),(C_{\mathrm{ME}},P_{\mathrm{ME}},F_{\mathrm{ME}}) \big ]. \end{aligned}$$

Simulations outputs were analysed based on the following variables which are deemed the most significant to calculate the risk of developing T2D: Glucose Base Level (*GBL*, namely the fasting glucose concentration indicated $\gamma (t)$), Body Mass Index (*BMI* indicated $\beta (t)$), and Tumor Necrosis factor-$\alpha$ (*TNF* representing the level of systemic inflammation, indicated $\tau (t)$) as measured in the adipose tissue compartment. The dynamic of M-T2D starting from the initial condition ${\varvec{x}}$ generates a complete trajectory of the variables $\beta (t),\gamma (t)$ and $\tau (t)$ with a time resolution corresponding to ten seconds. Since we are interested in analysing the condition of the virtual subject weekly, these measures are taken over a time horizon of twenty six weeks of routinely and uninterrupted physical activity and diet as specified in ${\varvec{x}}$. Formally,

2 $$\begin{aligned} {\varvec{y}} (t) = \left[ \beta \left( t\right) , \gamma \left( t\right) , \tau \left( t\right) \right] \end{aligned}$$

where $$t=1,\dots , T$$, and $$T=26$$.

The set $\left\{ \left( {\varvec{x}}^{k}, {\varvec{y}}^{k}\left( 1\right) , \dots , {\varvec{y}}^{k}\left( T\right) \right) : k=1,\dots ,K \right\}$ is used as a training set for the development of an emulator able to recapitulate, given ${\varvec{x}}$, the whole trajectory of the computational model and, finally, to predict the risk of developing T2D over the time horizon *T*. In other words, our goal is to find a *statistical model* able to emulate the dynamics of  the *dependent variables *of M-T2D, namely ${\varvec{y}}\left( t\right)$ for $t=1,\dots ,T$, given a set of *regressors/predictors* ${\varvec{x}}$, that is, the initial conditions defining the virtual subject and its lifestyle.

## Method

In the present work, we deal with a multi-output dynamic simulation model, which, given a set of input, it generates the dynamics of a multivariate vector over a given time horizon. The approaches proposed in the statistical literature to emulate such simulation models are mainly four.

The first method considers a multivariate output emulator in which the elements of the output vector are the values of the simulation variable at different time points [25, 26]. Such an approach is computationally demanding because it requires the construction of a multivariate emulator whose dimension depends on the length of the time series. A pitfall of this method is that it does not exploit the dynamics of the simulated process while relying just on the initial condition. This approach does not fit well those cases in which the modelled process has a large variability as for instance in stochastic simulations.

The second approach is to combine statistical models to describe the time evolution and Gaussian processes to model innovation terms, namely the differences between the statistical model prediction and the observed values [27, 28]. Beyond the use of a few ordinary differential equations to simulate a process, this approach becomes practically unfeasible.

The third method is the *one step ahead emulator* that is based on the assumption that the dynamics of the output variable only depends on the previous time point (Markovian property) and on the input variables [29–31]. This approach is considered the most reliable when dealing with models based on differential equations because it embeds the dynamics of the process. However, the performance of this method is poor if the simulated process has a large degree of stochasticity.

The fourth method considers time as an additional input variable. Also, this approach generally leads to a high computational cost of the emulator, particularly if Bayesian Gaussian processes are used, since it depends on the length of the time series [25, 32]. However, this approach turns out to be the most suitable for us because it allows constructing a multivariate emulator which accounts for the time dependency of the output and its stochasticity.

Considering the model, adding the time to the set of input variables translates into a very long dataset needed to train the model. More in details, the length of the dataset becomes $N=K\cdot T$, where *K* is the number of simulated trajectories and $T=26$ is the length of the output dynamics. In this case the number of input variables is eleven, namely, ten variables of Eq. (1) plus the time. One of the most successful models for these kinds of dataset, namely datasets which have a moderate number of features and a huge number of items, is the random forest [33]), which has already shown great fitting performance in [10]. Therefore we construct our emulator using a random forest algorithm in the last of the four methodologies briefly described above [25].

In detail, the proposed emulator can be mathematically described by the following formula

3 $$\begin{aligned} {\varvec{y}}\left( {\varvec{x}}, t\right) =\frac{1}{M}\sum _{m=1}^{M}{\mathcal {T}}_{m}\left( {\varvec{x}}, t\right) +\varvec{\epsilon }_{t} \end{aligned}$$

where ${\varvec{y}}\left( \cdot \right)$ is the output vector defined in equation (2), ${\mathcal {T}}_{m}\left( \cdot \right)$ is the *m*-th tree of the forest built on input $\left( {\varvec{x}}, t\right)$, ${\varvec{x}}$ is the vector of regressors defined in Eq. (1), *t* is a scalar that identify the time and $\varvec{\epsilon }\sim {\mathcal {N}}\left( {\varvec{0}}, \varvec{\Sigma }\right)$ represents the Gaussian error component.

The choice of random forest as learning algorithm, is a trade-off among interpretability of the results, fitting performance, reliability and computational efficiency handling large dimensional training set.

Other suitable machine learning algorithms are Support Vector Regression and Artificial Neural Network but they do not provide easily interpretable outcomes. Moreover, the former usually involves the choice of many parameters that strongly influence its performance. Gaussian Processes is another candidate but has a poor computational performance on large datasets.

The performances of the emulator have been analysed by considering different sizes for the training sets, namely $5\times 10^3$, $10\times 10^3$, $15\times 10^3$, and $20\times 10^3$. The training sets are subsets of the synthetic dataset containing $46170{}$ trajectories and they have been selected using a Latin hypercube sample schemes [34]. Such sample scheme is the most efficient sampling scheme, indeed, given a sample size it allows to select the most representative sample of that size. Each dataset considered has been divided in *training* and *test set* using the common proportion 70–30%. We considered $m=50$ trees which turned out to be a good choice while looking at the Out-Of-Bag error. For each trajectory we provide the predicted trajectory and a measure of uncertainty through the construction of bootstrap confidence bands, [35]. More in details, bootstrap confidence intervals are constructed through a modified version of the bootstrap samples, namely *bootstrapping residuals*, since we deals with regression models in which the regressors matrix is fixed. Bootstrapping consists of several iterations of resampling with replacement. Given the model prediction of the *i*-th trajectory, obtained as

$$\begin{aligned} \hat{{\varvec{y}}}\left( {\varvec{x}}_{i}, t\right) =\frac{1}{M}\sum _{m=1}^{M}\hat{{\mathcal {T}}}_{m}\left( {\varvec{x}}_{i}, t\right) \end{aligned}$$

the residuals are defined as

4 $$\begin{aligned} {\varvec{e}}\left( {\varvec{x}}_{i},t\right) = {\varvec{y}}\left( {\varvec{x}}_{i},t\right) -\hat{{\varvec{y}}}\left( {\varvec{x}}_{i},t\right) . \end{aligned}$$

We consider $B=50$ bootstrap samples of the residuals, then the bootstrap samples $\left\{ {\varvec{x}}_{i}, {\varvec{y}}^{\star }_{b}\left( {\varvec{x}}_{i},1\right) , \dots , {\varvec{y}}^{\star }_{b}\left( {\varvec{x}}_{i},T\right) \right\} _{i=1}^{n}$ for $b=1,\dots , B$ are obtained as

$$\begin{aligned} {\varvec{y}}^{\star }_{b}\left( {\varvec{x}}_{i},t\right) = \hat{{\varvec{y}}}\left( {\varvec{x}}_{i},t\right) +{\varvec{e}}^{\star }_{b}\left( {\varvec{x}}_{i},t\right) \end{aligned}$$

where ${\varvec{e}}^{\star }_{b}\left( {\varvec{x}}_{i},t\right)$ is the *i*-th residual at time t of the *b*-th bootstrap sample. The bootstrap samples provide *B* predictions, one for each trajectory from which it is possible to compute statistics such as variance, confidence intervals, quantiles, etc. Further details on the bootstrap technique can be found in [35].

## Results

The emulation method has been implemented in R language. Its performance has been evaluated by considering the Root Mean Square Error, *E* defined for each output variable at time *t* as

5 $$\begin{aligned} E^{h}\left( t\right) = \sqrt{\frac{1}{n}\sum _{i=1}^{n}\left( y^{h} \left( {\varvec{x}}_{i},t\right) -{\hat{y}}^{h}\left( {\varvec{x}}_{i},t \right) \right) ^2} \end{aligned}$$

where $h\in \left\{ \beta , \gamma , \tau \right\}$ is the index which refers to one of the output variable, $y^{h}\left( {\varvec{x}}_{i},t\right)$ refers to the *i*-th trajectory of the dependent variable defined by *h* at time $t=1,\dots , 26$, ${\hat{y}}^{h}\left( {\varvec{x}}_{i},t\right)$ is the corresponding fitted observation. By definition, *E* is the standard deviation of the residuals defined in Eq. (4), therefore it measures the accuracy of the model. Since the value of *E* depends on the variables scale, we rescale the output variables codomain to the unit interval $\left[ 0,1\right]$; this allows immediate comparison of the performance of the model among the output variables: the closer *E* to zero the better the model accuracy. The computational time the emulator requires to be trained depends on the size of the training set. To give an idea, the emulator requires less than 5 min to be trained with a dataset of 17.500 observations on a common single processor computer.

**Fig. 1:**
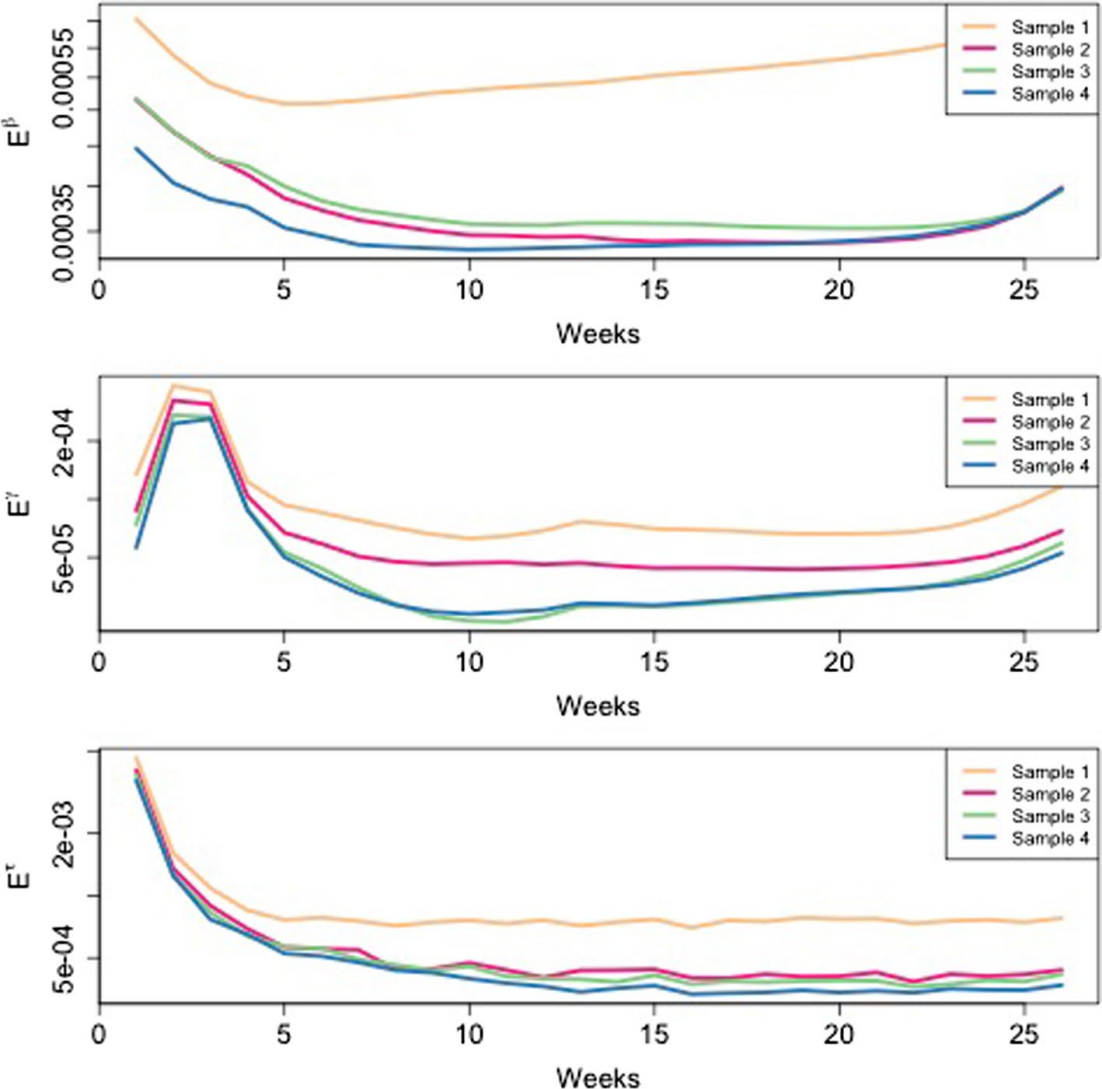
Root mean square error (E) for the $\beta$ (top panel), $\gamma$ (middle panel) and $\tau$ (bottom panel). The *x*-axis represents the time of the trajectories in weeks. Values of E closer to zero mean better predictions. Each plot contains the performance related to each sample considered. Sample 1 contains 5000 observation, Sample 2 contains 10000 observations, Sample 3 contains 15000 observation and Sample 4 contains 20000

**Fig. 2:**
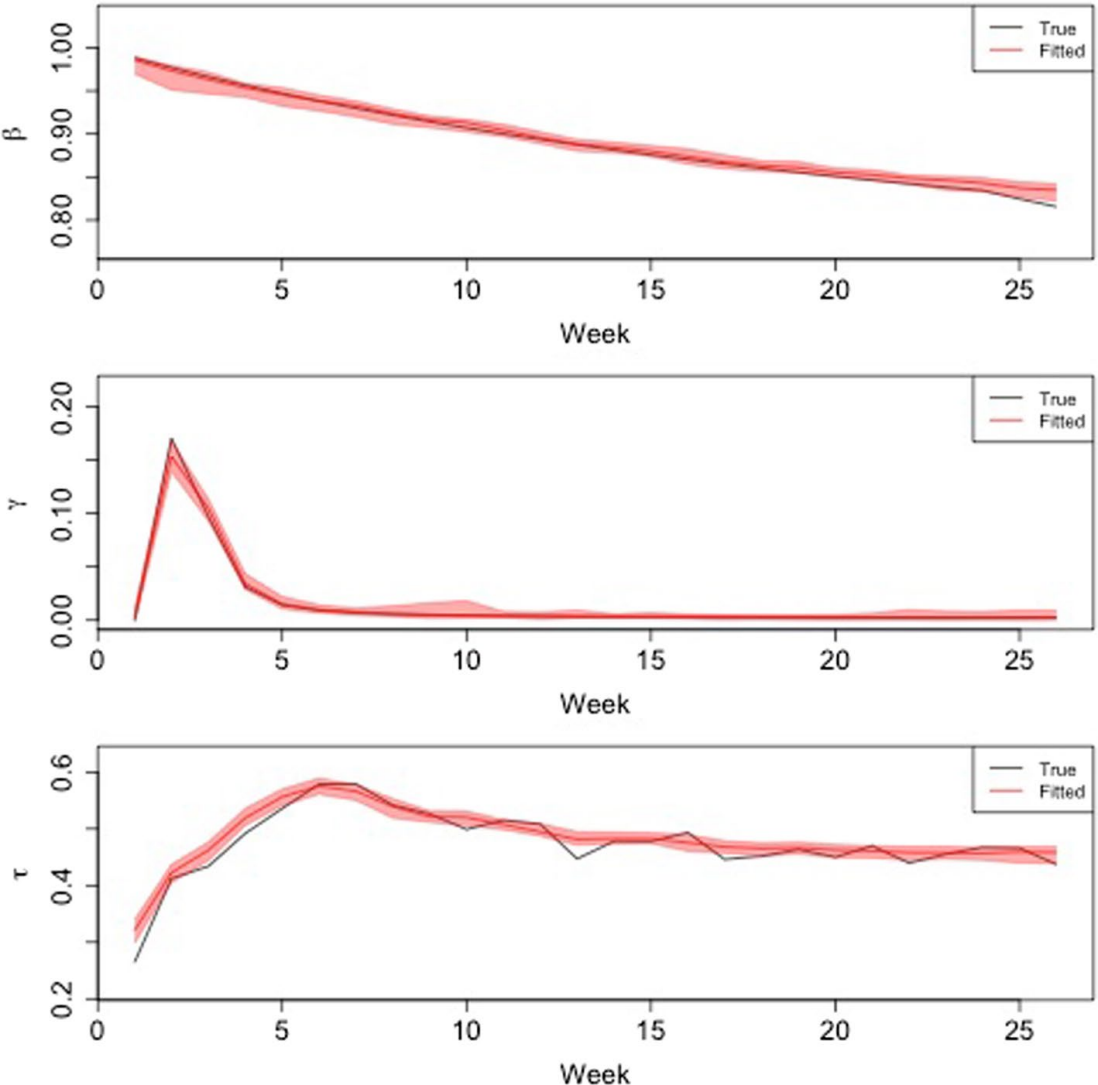
Dynamics of the true output variable versus the fitted one together with the $95\%$ bootstrap confidence interval. On the *x*-axis there is the time of the trajectories in weeks while on the *y*-axis the output variable normalised to 0–1. The first panel from the top that refers to $\beta (t)$ shows a good fitting precision as the true trajectory always falls inside the confidence band. The second panel refers to $\gamma (t)$ and it shows that again the emulator shows good fitting performance The third panel refers to $\tau (t)$ and shows that while the emulator is able to catch the overall trajectory, due to the high variability of the true trajectory, the smoother confidence band does not embed it completely

**Fig. 3:**
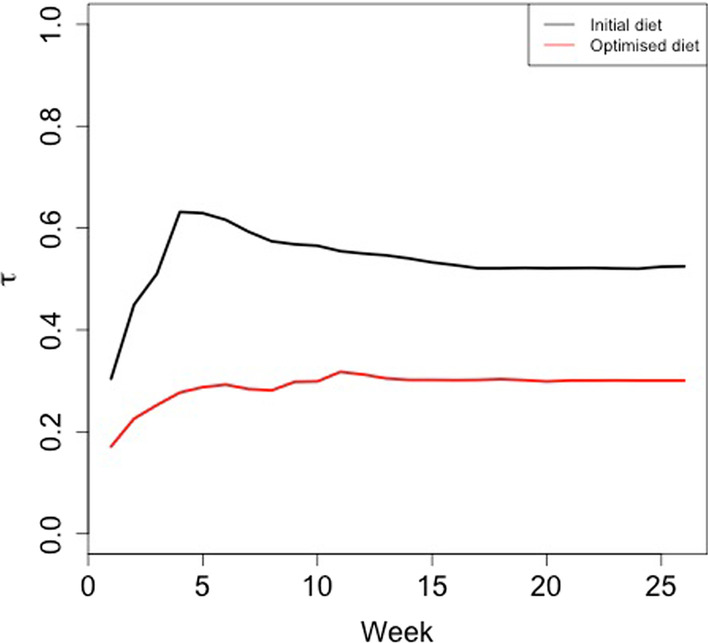
The plot shows $\tau$ related to a 38 years old overweight male of average height, who exercises once a week for 30 minutes with an intensity of 60%VO_2max_. The black line shows $\tau$ with the actual medium carb and prot-fat rich diet while the red line is the result of an optimised diet consisting of low carb and fat while rich in protein

In Fig. 1 the dynamics of the *E*s for samples of different sizes have been shown. The glucose base level $\gamma \left( t\right)$ is the variable that shows better fitting performance since $E^{\gamma }$ is always smaller than $E^{\beta }$ and $E^{\tau }$ for each sample considered.

Looking at the behaviour of the *E*s it turns out that the emulator has more difficulties in predicting the first and the last periods, while in between it shows better performances.

In [12] we considered the one-step-ahead emulator method, whose performances are lower than those presented here. Indeed, the values of *E*s for the one step ahead emulator are between $10^{-2}$ and $2\times 10^{-1}$ for each output variable while for the methodology considered in this paper the values of *E*s are between $5\times 10^{-5}$ and $7\times 10^{-3}$. In other words there is an improvement in terms of *E*s of two orders of magnitude. Moreover, the dynamics of the *E*s are very different between the two approaches, indeed in the one-step-ahead emulator the dynamics of *E*s are always increasing for $\beta \left( t\right)$ and $\gamma \left( t\right)$ while it is almost stationary for the $\tau \left( t\right)$. This behaviour suggests a structural bias in the emulator. Instead, the dynamics of the *E*s reported here have U-shape and, as already pointed out, much lower values which do not reflect any structural bias.

The Root Mean Square Error provides an overall measure of the performance of the emulator for each time point, but it does not help in identifying the area of the domain that is more difficult to emulate. For this reason, the performance of the emulator has been tested on each trajectory by using the bootstrap confidence bands method. Specifically, for each trajectory we got 50 bootstrap predictions and the confidence bands have been constructed by selecting the quantiles $q_{\alpha }$ and $q_{1-\alpha }$ for $\alpha =0.05$ for each time point $t=1, \dots , T$. In Fig. 2 we show an example of the bootstrap confidence band constructed for a given trajectory. Each panel refers to an output variable. The results for the variables $\beta \left( t\right)$ (Fig. 2 top panel) and $\gamma \left( t\right)$ (Fig. 2 middle panel) are satisfactory because the simulation trajectory always falls inside it.

The confidence band of the $\tau \left( t\right)$ follows the dynamics of the variable but sometimes the reference trajectory falls outside it. This is due to the fact that the emulator reproduces the average behaviour of the output variable providing therefore a smoother trajectory. The higher variability of $\tau \left( t\right)$ compared to $\gamma \left( t\right)$ and $\beta \left( t\right)$ is mainly due to the considerably shorter life span of cytokines (such as the Tumor Necrosis Factor) when compared to the coarse grain of the time observations.

To have a general idea of the performance of the bootstrap confidence bands, the fraction of time in which the reference trajectory falls inside the confidence band (what is called the coverage probability) has been reported in Table 2. As expected, the coverage probability increases with the dimension of the sample size and, according to what already observed in Fig. 2, $\beta \left( t\right)$ and $\gamma \left( t\right)$ have a satisfactory coverage probability while $\tau \left( t\right)$ has worse coverage probability due to its intrinsic variability.

## Controlling the inflammation level

The excess of calories has a direct influence on the state of inflammation [19], which, in turn, can be monitored through the Tumor Necrosis Factor cytokine. The aim of this section is to describe how the emulator can be used to solve the optimisation problem arising in controlling the inflammation (i.e., the Tumor Necrosis Factor) through the diet. Specifically, given the initial condition representing a virtual individual $(S, A, W, H, N_{\mathrm{PA}}, D_{\mathrm{PA}}, I_{\mathrm{PA}}, C_{\mathrm{ME}}, P_{\mathrm{ME}}, F_{\mathrm{ME}})$, the goal is to keep Tumor Necrosis Factor (i.e., $\tau \left( t\right)$) under control by tuning the calorie intake in terms of carbs, proteins and fats.

The translation of this optimisation problem into a mathematical framework requires the knowledge of the variables domain, in other words, we need to know if the variable to be optimised has some constraint.

The quantities of carbohydrates, proteins, and fats are computed in the in M-T2D taking into account the balance of calories between the meal and the total daily energy expenditure (TDEE) [16]. In details, the TDEE is the result of the sum of Resting Energy Expenditure (REE), Activity Energy Expenditure (AEE) [36] and Thermic Effect of Food (TEF) [37]. The REE has been computed considering *S*, *A*, *W* and *H* [17]; the computation of AEE is based on $N_{\mathrm{PA}}, D_{\mathrm{PA}}$ and $I_{\mathrm{PA}}$, [16]; the TEF is the amount of energy spent to digest the food and amounts to about 10% of the calories ingested [37]. The resulting TDEE represents the number of calories that have to be ingested to have a balance among energy intake and expenditure. The simulation trajectories have been constructed by dividing the TDEE into three meals and the caloric content of each meal has been divided into calories from carbohydrates, proteins, and fats according to the kind of *standard proportions* 50%, 20%, and 30%, respectively. Simple multiplications to the constants 0.8 and 1.5 are used to fix *low* and *high* quantities of the food intake description given in Table 1.

The optimisation problem we want to solve can be detailed as follows. Given an initial vector ${\varvec{x}}$ as described in Eq. (1) we minimise $\tau \left( t\right)$ by modifying $C_{\mathrm{ME}}$, $P_{\mathrm{ME}}$, and $F_{\mathrm{ME}}$ subject to some constraint, that is,

$$\begin{aligned}{}&\min _{C_{\mathrm{ME}},P_{\mathrm{ME}},F_{\mathrm{ME}}}\sum _{t}\tau \left( t\right) ^2\\&s.t. \ \ \small {l_{C}\le C_{\mathrm{ME}}\le u_{C}}\\&\qquad \small {l_{P}\le P_{\mathrm{ME}}\le u_{P}}\\&\qquad \small {l_{F}\le F_{\mathrm{ME}}\le u_{F}}\\&\qquad \small {l_{T}\le C_{\mathrm{ME}}+P_{\mathrm{ME}}+F_{\mathrm{ME}}\le u_{T}} \end{aligned}$$

where $l_{C}, l_{P},l_{F}$ and $u_{C}, u_{P},u_{F}$ represent respectively the lower and upper bounds for the amount of carbs, proteins and fats for meal related to the individual and obtained according to the previous description while $l_{T}$ and $u_{T}$ represent the lower and upper total amount of food, that is $C_{\mathrm{ME}}+P_{\mathrm{ME}}+F_{\mathrm{ME}}$.

Given the computational complexity of the simulation M-T2D, solving this mathematical optimisation problem (whatever the algorithm used) would require M-T2D to run for each optimisation step and therefore would take considerable time to converge. By using the emulator instead, the problem becomes much easier computationally since the emulator is much quicker to run.

In Fig. 3 we show an example of the optimisation obtained for an individual having the following features: $S=man$, $A=38$, $H=average$, $W=overweight$, $N_{PA}=1$, $D_{PA}=30$, $I_{PA}=60$, $C_{ME}=Medium$, $P_{ME}=High$ and $F_{ME}=High$. The black line in Fig. 3 shows $\tau (t)$ according to these initial conditions. We run the optimisation to suggest a suitable (and patient-specific) diet, whose aim is to keep the $\tau (t)$ at the lowest level possible. In Fig. 3 the red line shows $\tau (t)$ corresponding to the optimal protein rich diet (i.e., low in carbs and fats).

## Discussions

Although Gaussian Process is the most used statistical model for emulation purpose, its computational cost become unfeasible when dealing with dynamic and multi-output simulators which require thousands of trajectories to be trained. In such situations machine learning algorithms are satisfactory alternatives. Indeed, the use of machine learning algorithms with bootstrap methods provides a set of information which is quite complete, namely statistics which provide information regarding estimates uncertainty, even if it is not detailed as the one provided by Gaussian processes, which instead provide the entire distribution of the parameters and therefore full information regarding estimates uncertainty.

This work shows that random forest has high performances as emulator of a complex biological simulator. This is due to the fact that random forest is particularly suited for agent-based models and for long data sets, namely data sets which have a moderate number of features and a huge number of instances, which is the type of data set we are dealing with in this work. Of course, a comparison with several machine learning algorithms would be very interesting, although out of scope of this work, but it will be addressed in future works.

The choice to add the time variable within the regressors, although increases the number of instances, allows to jointly estimates the three output variable using all the information available in the data set. This is worthwhile because the three variables have different time of reaction as observed in Fig. 2 which shows the kind-of inertia the immune inflammation has, namely the *memory* effects of the adaptive immune response. In other words there is lag between a decrease of $\beta$ and $\gamma$ and a decrease of $\tau$ just because the resolution of the inflammation takes more time with respect to a reduction in glucose base level (which has a faster dynamics) and a reduction of the BMI. Moreover, worth to note, the peak of the curve $\tau$ is due to the sudden peak of $\gamma$ while the pretty slow decline follows the decline of $\beta$ combined with the inertia of the overall immune response.

The high accuracy of the proposed emulator allows to perform an optimisation problem aimed at controlling the inflammation while leveraging on the variables related to individual patient diet. In other words, it is possible to perform quick-and-easy self-monitoring assessments and also to provide personalised suggestions regarding dietary habits which help in keeping the inflammation at low level. The optimisation can be designed in order to consider other variables to be optimised, for instance it is possible to minimise the Tumor Necrosis Factor-$\alpha$ while leveraging on the variables related to physical activity or to diet and physical activity, in order to provide suggestions regarding dietary habits and/or physical activity.

The possibility to emulate complex biological model so to be executed in real-time and on devices having limited computational power is worthwhile in the context of personalised medicine. Indeed, the output of the emulator and its applications can support medical staff in decision and interventions since it provides information which are tailored on individual patients. Moreover, its value is also related to the development of self-monitoring systems nowadays embedded in portable communication devices which opens up to the application of predictive tools in health care.

## Conclusions

Computer simulation is a fundamental instrument in virtually all scientific research fields including medicine and biology where in-vitro or in-vivo experiments are often unfeasible for economic and ethical reasons. Computer models offer a valid alternative to realistic experiments but might nevertheless be computationally expensive, especially when the details and the precision required is high. The computational cost of these models makes real-time execution of the simulations on mobile devices unfeasible in practice. In this regards the development of emulators in place of heavy simulations constitutes a viable alternative for the implementation of self-monitoring devices due to their reduced computational needs.

This paper deals with the construction of an emulator of a validated multi-level patient-specific simulation model based on machine learning techniques, which turn out to have good performances although the complexity of the simulation model being a mixture of agent-based models and systems of differential equations. This emulator can be used to solve optimisation problem required to perform quick-and-easy self-monitoring assessments. For instance, in this work it has been used to compute personalised diets able to reduce or to keep inflammation at low levels.

To summarise, the implementation of this predictive model on self-monitoring devices is worthwhile given the incidence of type-2 diabetes, which account for 85–90% of all cases of diabetes in the World [38], and it justifies the importance of having an emulator with optimal fitting performance.

## Data Availability

The datasets generated and/or analysed during the current study are available as a web-service at the following address: http://kraken.iac.rm.cnr.it/T2DM.

## Abbreviations

T2D: Type 2 diabetes
M-T2D: Multi-scale immune system simulator for the onset of type 2 diabetes
BMI: Body mass index
GBL: Glucose base line
TNF-$\alpha$: Tumor necrosis factor-$\alpha$
ML: Machine learning
TDEE: Total daily energy expenditure
REE: Resting energy expenditure
TEF: Thermic effect of food
AEE: Activity energy expenditure
S: Sex
A: Age
W: Weight
H: height
$\hbox {N}_{{PA}}$: Number of physical activity sessions per week
$\hbox {D}_{{PA}}$: Duration of physical activity sessions in minutes
$\hbox {I}_{{PA}}$: Intensity of physical activity sessions
$\hbox {C}_{{ME}}$: Mean carbohydrates intake per day
$\hbox {F}_{{ME}}$: Mean fat intake per day
$\hbox {P}_{{ME}}$: Mean protein intake per day
*E*: Root mean square error
